# Combination of Polysaccharide Nanofibers Derived from Cellulose and Chitin Promotes the Adhesion, Migration and Proliferation of Mouse Fibroblast Cells

**DOI:** 10.3390/nano12030402

**Published:** 2022-01-26

**Authors:** Tomoka Noda, Mayumi Hatakeyama, Takuya Kitaoka

**Affiliations:** Department of Agro-Environmental Sciences, Graduate School of Bioresource and Bioenvironmental Sciences, Kyushu University, Fukuoka 819-0395, Japan; t.noda@agr.kyushu-u.ac.jp (T.N.); m_hatakeyama@agr.kyushu-u.ac.jp (M.H.)

**Keywords:** cellulose nanofiber, chitin nanofiber, surface carboxylation, surface deacetylation, cell culture scaffold, skin repair, wound healing, biomedical applications

## Abstract

Extracellular matrix (ECM) as a structural and biochemical scaffold to surrounding cells plays significant roles in cell adhesion, migration, proliferation and differentiation. Herein, we show the novel combination of TEMPO-oxidized cellulose nanofiber (TOCNF) and surface-*N*-deacetylated chitin nanofiber (SDCtNF), respectively, having carboxylate and amine groups on each crystalline surface, for mouse fibroblast cell culture. The TOCNF/SDCtNF composite scaffolds demonstrated characteristic cellular behavior, strongly depending on the molar ratios of carboxylates and amines of polysaccharide NFs. Pure TOCNF substrate exhibited good cell attachment, although intact carboxylate-free CNF made no contribution to cell adhesion. By contrast, pure SDCtNF induced crucial cell aggregation to form spheroids; nevertheless, the combination of TOCNF and SDCtNF enhanced cell attachment and subsequent proliferation. Molecular blend of carboxymethylcellulose and acid-soluble chitosan made nearly no contribution to cell culture behavior. The wound healing assay revealed that the polysaccharide combination markedly promoted skin repair for wound healing. Both of TOCNF and SDCtNF possessed rigid nanofiber nanoarchitectures with native crystalline forms and regularly-repeated functional groups, of which such structural characteristics would provide a potential for developing cell culture scaffolds having ECM functions, possibly promoting good cellular adhesion, migration and growth in the designated cellular microenvironments.

## 1. Introduction

An extracellular matrix (ECM) is a non-cellular biocomponent filled up in the intercellular spaces within tissues and organs, which is well known as structural and biochemical scaffolding constituents, such as collagen with a rigid nanofibrous protein, proteoglycans composed of core proteins and glycosaminoglycan chains, and a linear polysaccharide, e.g., hyaluronan [1]. These biological components found in vivo provide the structural frameworks for cell adhesion and growth, subsequently affecting proliferation and differentiation of the attached cells [2]. In recent years, the development of cell culture scaffolds that mimic the ECM components and cell-surrounding microenvironments has been actively carried out [3]. At the beginning, intact collagen from animal sources was used to promote cell attachment and angiogenesis in tissue engineering [4]. In a similar way, natural hyaluronan was also effective for wound healing when cultured with chondrocytes around damaged human cartilages [5]. However, such ECM components are often derived from animal origins, resulting in a risk of various infectious diseases and immune responses [6]. Furthermore, the extracted components are crude mixtures whose quality at chemistry level is not always constant; therefore, the lot-to-lot variation critically reduces reproducibility for expected performances. For that reason, novel xenobiotic but biocompatible components with various ECM functions have been required to design bioadaptive cell culture scaffolds, and in that case structurally-defined rigid nanofibers are promising candidates for this purpose.

Cellulose, the major structural component of wood cell walls, is the most abundant biopolymer on the earth, which forms crystalline rigid nanofibers composed of dozens of linear *β*-1,4-linked D-glucopyranose chains assembled by intra- and inter-molecular hydrogen bonding [7,8]. In recent years, cellulose nanofibers (CNFs) have attracted much attention in academic and industrial circles due to their high strength, light weight, high transparency, low thermal expansion and various fascinating features [9,10,11]. Most of CNFs can be isolated from wood and pulp fibers by physicochemical downsizing to a nanometer level. One approach to obtain CNFs is an aqueous catalytic oxidation of cellulose sources with 2,2,6,6-tetramethylpiperidine 1-oxyl (TEMPO) [12]. The TEMPO-mediated oxidation is allowed to site-selectively convert hydroxymethyl group to carboxylate one at the C6-position exposed on the crystalline surface of cellulose microfibrils [13]. The TEMPO-oxidized cellulose nanofiber (TOCNF) has a unique core–shell structure with intact cellulose in the core and polyuronate in the shell, and thus it is one of the most promising nanomaterials in eco-friendly and sustainable industries. Chitin (Ct) is also a natural biopolymer commonly found in shells of marine crustaceans and cell walls of fungi [14]. It is a typical liner polysaccharide, composed of *N-*acetyl D-glucosamine linked only in a *β*-1,4 manner like cellulose. Chitosan (Cs), a deacetylated derivative of Ct, is a positively-charged natural polymer that has unique bioactive properties such as antibacterial activity [15], wound healing [16] and analgesic effects [17]. Lately, chitin nanofiber (CtNF) [18], chitosan nanofiber (CsNF) [18] and surface *N*-deacetylated CtNF (SDCtNF) [19], which are obtained by nano-pulverization and stepwise deacetylation, are expected in the biomedical applications because of their structural properties as well as CNFs [20]. Especially, SDCtNF also has a unique core–shell structure with intact Ct in the core and Cs in the shell, similar to the nanoarchitecture of the TOCNF [19].

A variety of cell culture scaffolds composed of polysaccharides have been actively investigated [21,22,23]. An attempt on the molecular blend of Cs and hyaluronan was carried out to fabricate hybrid fibers as a scaffolding biomaterial for cartilage tissue engineering [24], on which chondrocytes well proliferated while maintaining their morphological phenotype and producing ECMs, especially type II collagen, around the cells. In addition, Cs and collagen composites have been investigated to promote osteoblast proliferation, differentiation and matrix mineralization for MC3T3-E1 cell culture [25]. Besides, such combination drastically increased transcriptional activity of Runx2, which is an important factor to regulate the downstream of osteoblast differentiation of phosphorylated Erk1/2 [25]. Our previous works have reported that glyco-biointerfaces composed both of chitohexaose (*β*GlcNAc6) and cellohexaose (*β*Glc6) promoted the activation of a specific detoxification enzyme of human hepatocellular carcinoma (HepG2) cells [26]. Furthermore, we unveiled that the inflammatory response of human embryonic kidney (HEK293) cells strongly depended on the surface *β*GlcNAc6 density, via direct stimulation triggered by toll-like receptor 2 [27]. However, neither molecular blends nor oligosaccharides assembly have any nanofiber structures, which are found in vivo around cells. TOCNF-containing hydrogels showed non-cytotoxicity for rat bone marrow-derived mesenchymal stem cells, and L929 fibroblast cells in vitro, and the hydrogels significantly reduced peritoneal adhesion in rats compared to untreated controls by in vivo evaluations [28]. Besides, in recent years, we have reported the carboxylate content-dependent cell proliferation of mouse fibroblasts by using TOCNF-based cell culture scaffolds, on which cellular behavior varied according to the surface physicochemistry [29]. Thus, rigid polysaccharide nanofibers must have the potential to develop a new type of bioadaptive cell culture scaffolds.

**Figure 1:**
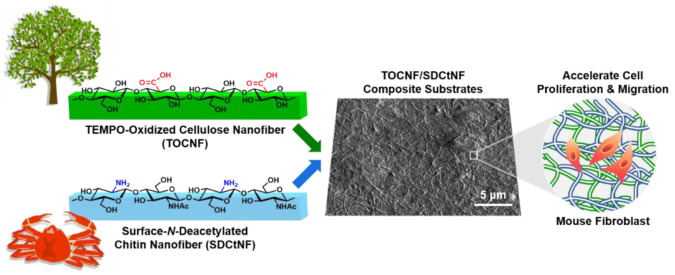
Schematic illustration of the research strategy in this work for the combination of TEMPO-oxidized cellulose nanofiber (TOCNF) and surface *N*-deacetylated chitin nanofiber (SDCtNF) to form crystalline-nanofibers-based cell culture scaffolds.

In this work, the novel combination of crystalline TOCNF and SDCtNF having carboxylate and primary amine groups, respectively, on each nanofiber surface was first investigated to develop bioadaptive cell culture scaffolds, as illustrated in Figure 1. We aimed at elucidating the characteristic cellular behavior induced by mixing polysaccharide NFs with different ratios and providing a potential to use these structurally-defined polysaccharide NFs as a new candidate for developing cell culture scaffolds. Rigid nanofibers and repeated functional groups of nano-polysaccharides demonstrated the unique cell attachment, migration and proliferation behavior of mouse fibroblast cells, although molecular blending of water-soluble polysaccharides analogous to the nanofibers used was ineffective. Our strategy is expected to provide a new insight into biomaterials design in cell culture engineering.

## 2. Experimental

### 2.1. Materials

TOCNF (0.93 wt% aqueous suspension, COOH: 1.59 mmol g^–1^) and SDCtNF (1.0 wt% aqueous suspension, NH_2_: 1.71 mmol g^–1^) were kindly provided, respectively, by Nippon Paper Industries Co., Ltd., Tokyo, Japan and Marine Nano-fiber Co., Ltd., Tottori, Japan. Mouse fibroblast-like cell line (NIH/3T3) was purchased from RIKEN BRC, Tsukuba, Japan, through the National BioResource Project of the MEXT/AMED. Dulbecco’s modified Eagle’s medium (DMEM, high glucose), L-glutamine, penicillin–streptomycin, sodium pyruvate solution, and trypsin–ethylenediaminetetraacetic acid (EDTA) were obtained from Life Technologies Co., Carlsbad, CA, USA. Fetal bovine serum (FBS) was received from Biowest Co., Ltd., France. Micro-cover glass (diameter: 15 mm, Matsunami Glass Ind. Ltd., Osaka, Japan) was used as a base material to form polysaccharide cast films. Tissue culture polystyrene (TCPS) 24-well plates (Sumitomo Bakelite Co., Ltd., Tokyo, Japan) was used as cell culture substrate. Calcein AM (4 mM DMSO solution) for green fluorescent staining of live cells, and ethidium homodimer III (2 mM DMSO/H_2_O 1:4 *v*/*v* solution) for fluorescent staining in red of dead cells were purchased from PromoCell GmbH, Germany (Live/Dead Cell Staining Kit II). Cell Counting Kit-8 (CCK-8; Dojindo Laboratories Ltd., Kumamoto, Japan) was used to measure the numbers of living cells. The water used in this study was purified with a Barnstead Smart2Pure system (Thermo Scientific Co., Ltd., Japan). Other reagents were of a special grade and used as received without further purification.

### 2.2. Preparation of Cell Culture Scaffolds from Polysaccharide Nanofibers

Each TOCNF or SDCtNF aqueous suspension was re-suspended to set at 0.4 wt% by adding purified water. The aqueous suspensions of nanofibers, TOCNF alone, SDCtNF alone, and the mixtures of TOCNF and SDCtNF with a molar ratio of COOH:NH_2_ = 1:1, 2:1 and 4:1, were prepared. Each suspension was sufficiently dispersed to afford clear suspension with the use of an ultrasonic homogenizer (Ultra homogenizer VP-5S, TAITEC Co., Koshigaya, Japan) for 30 s, and casted on a micro-cover glass in an amount of 200 µL (nanofiber content: 0.8 mg in total), followed by being air-dried overnight at room temperature. Table S1 lists the actual amounts of each nanofiber coated on the micro-cover glass. The dried substrates were sterilized by immersion in ethanol with UV light for 20 min inside a clean bench.

### 2.3. Characterization of Polysaccharide Nanofibers and Substrates

The morphology of polysaccharide nanofibers used in this study, TOCNF, SDCtNF and TOCNF/SDCtNF, was observed by a transmission electron microscope (TEM; JEM-2100HC, JEOL Ltd., Tokyo, Japan) at an accelerating voltage of 200 kV. The diluted aqueous suspension was dropped onto a copper grid (elastic carbon coated, ELS-C10 STEM Cu100P grid specification, Ohken Shoji Co., Ltd., Tokyo, Japan), and then dyed with 1% sodium phosphotungstate for 3 min before the TEM observation. The surface nanotopography of the substrates was observed at room temperature in air using an atomic force microscope (AFM, Dimension Icon, Bruker Japan Co., Ltd., Tokyo, Japan) at a peak force tapping (ScanAsyst) mode. The measurement was performed with a scanning range of 20 × 20 µm^2^ using a silicon nitride probe. Root mean square roughness (*R*a) was calculated from obtained AFM images. The crystalline structures of freeze-dried samples were analyzed using an X-ray diffractometer (XRD; SmartLab, Rigaku Co., Ltd., Tokyo, Japan) with Cu Kα radiation (λ = 0.15418 nm). The XRD patterns were recorded at 40 kV within a scan range of 5° to 40° and a scan rate of 2° per min. The crystallinity indices were calculated in accordance with the Segal method [30]. Optical images of the coated films were taken by a digital camera. The surface wettability was analyzed by the sessile drop method using a contact angle meter (DropMaster 500, Kyowa Interface Science Co., Ltd., Niiza, Japan).

### 2.4. Cell Culture and Counting

Mouse fibroblast NIH/3T3 cells were cultured in DMEM supplemented with FBS (10%, *v*/*v*), penicillin–streptomycin (100 U and 100 μg mL^–1^, respectively) at 37 °C in a humidified atmosphere of 5% CO_2_ and 95% air. Each sterilized substrate was gently placed at the bottom of commercial 24-well TCPS plates with the top inner and bottom inner diameters of 16.3 mm and 15.1 mm, respectively. A total of 500 µL of cell suspension (1.0 × 10^5^ cells mL^–1^) was seeded on each substrate, and cultured at 37 °C. After incubation for 24, 48 and 72 h, the cultured cells were observed with a Leica DMI 4000B microscope (Leica Microsystems GmbH, Wetzlar, Germany). The number of living cells was measured by CCK-8 assay. Prior to cell counting, CCK-8 solution was added to each well by 25 µL, and incubated for 1.5 h (37 °C and 5% CO_2_). After 1.5 h incubation, 200 µL solution was transferred from each well to a 96-well plate, and then absorbance at 450 nm was measured using a microplate reader (iMark microplate reader, Bio-Rad Laboratories Inc., Hercules, CA, USA). For the obtained absorbance, the number of living cells was quantified using a calibration curve made in advance.

### 2.5. Cell Assays

Cell viability on each substrate was confirmed by fluorescence observation after cell staining. After removing the medium from each well and washing with 1 mL of phosphate buffered saline (PBS), 200 µL of the staining solution containing 2 µM calcein AM and 4 µM ethidium homodimer III solutions in PBS, were added to each well, and then incubated for 30 min at 37 °C and 5% CO_2_. Live cells were stained in green, and dead ones in red. Wound healing assay was carried out using Culture-Insert 2 Well (ibidi GmbH, Germany) according to the manufacturer’s instruction. In brief, the Culture-Insert 2 Well having a 500-µm thickness was placed on the polysaccharide NFs-coated surface, while preventing any cell growth beneath the insert. NIH/3T3 cells were seeded on the separated two reservoirs in the well at a density of 2.8 × 10^4^ cells per each reservoir. After allowing the cells to attach overnight, the culture-insert was removed, followed by adding a fresh medium. It was confirmed that the polysaccharide NFs layers remained intact without peeling off after removing the culture insert. Width of cell-free gap was set at 500 ± 100 μm, and cell migration behavior was monitored over time using a digital microscope (WSL-1800 CytoWatcher, ATTO Co., Ltd., Tokyo, Japan). Cell-free gap areas were captured by Image J software (National Institutes of Health, Bethesda, MD, USA) with Wound Healing Size Tool (an ImageJ/Fiji plugin) [31]. The percentage of wound closure was expressed as coverage percentage of wound closure on an initial area basis, according to the following equation:

$$\text{Wound closure}\;(\%)=\left[\frac{\left(At_{0}-At\right)}{At_{0}}\right]\times 100$$

where *At*_0_ is the initial cell-free area at time 0, and *At* is the cell-free area observed at 4, 8, 12, 16, 20 and 24 h.

## 3. Results and Discussion

### 3.1. Structural Characteristics of Polysaccharide Nanofibers and the Substrates

**Figure 2:**
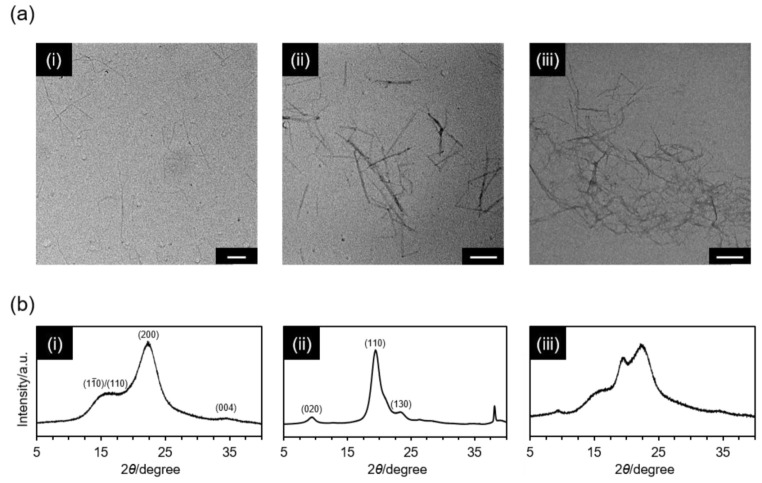
TEM images (**a**) and XRD patterns (**b**) of (**i**) TOCNF, (**ii**) SDCtNF and (**iii**) TOCNF/SDCtNF (COOH:NH_2_ = 4:1). Scale bars in (**a**) = 200 nm.

Nanoscale morphology of polysaccharide NFs used in this study was observed by TEM imaging. The obtained result clearly visualized independently-dispersed TOCNF and SDCtNF, having very thin fiber width, as shown in Figure 2a. Fiber width and ζ-potential values are listed in Table S1. Wood and crab-derived natural nanofibers are precisely constructed during biosynthesis, and nanomorphologies of TOCNF and SDCtNF corresponded well to the literature data [32]. Surface charges of TOCNF and SDCtNF at pH = 7.0 were ca. −46.8 mV and 17.5 mV, respectively, originating from dissociated caboxylate and protonated amine, while those of the mixtures depended on the molar ratios of COOH:NH_2_. In the case of TOCNF/SDCtNF mixture, a lot of entangled nanofibers were observed, albeit being indistinguishable from each other. The XRD patterns of TOCNF, SDCtNF and the mixture are depicted in Figure 2b. TOCNF exhibited four major peaks at 2θ  =  14.8°, 16.4°, 22.6° and 34.2°, corresponding to (1–10), (110), (200) and (004) crystal planes of native cellulose I, respectively [33,34]. SDCtNF exhibited two typical peaks at 2θ = 9° and 19°, corresponding to (020) and (110) crystal planes of α-chitin [35,36]. The crystallinity index of TOCNF was ca. 66.6%, while that of SDCtNF was ca. 97.3%. Both strongly indicated the crystalline cores of each nanofiber, on which carboxylate (1.59 mmol g^–1^) and amine (1.71 mmol g^–1^) groups were present only on the surfaces for TOCNF [13] and SDCtNF [19], respectively. Therefore, TOCNF and SDCtNF used in this study presumably possessed unique core–shell structures composed of crystalline cores and functionalized shells. These nanofibers were actually very thin as compared to the natural ECM components such as collagen microfibers found in vivo, while they appeared similar to tropocollagen with 1.5-nm diameter and 300-nm length in nanomorphology [37]. In this context, TOCNF and SDCtNF were not directly involved in acting as structural analogues to native ECM components. On the other hand, the nanometer-scale topography is considered to affect the intra-/intercellular sensing functions [38,39]; therefore, thin TOCNF and SDCtNF would be expected to assume some influence on the cell behavior at the biointerfaces.

**Figure 3:**
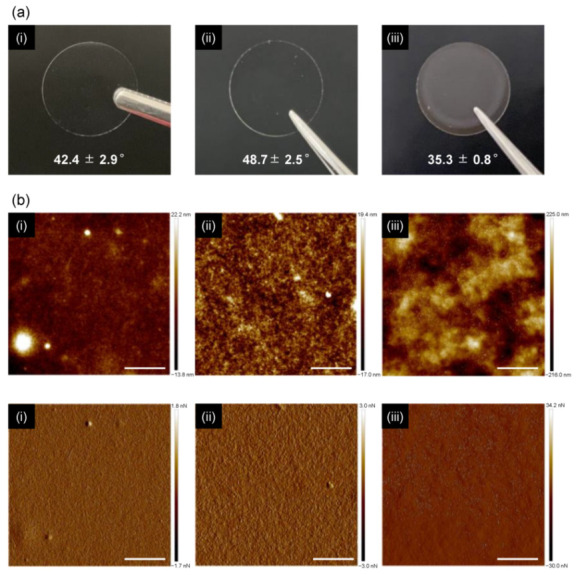
Optical images of polysaccharide NFs-coated glass substrates (**a**) and AFM images (**b**) of (**i**) TOCNF alone, (**ii**) SDCtNF alone and (**iii**) TOCNF/SDCtNF composite (COOH:NH_2_ = 4:1). Numerical values in the optical images are the contact angles of a water droplet on each substrate after sterilization. **Upper** and **lower** images of (**b**) correspond to the topological and peak force error images, respectively. Scale bars in (**b**) = 5 μm.

Optical images and water wettability of polysaccharide NFs-coated glass substrates are shown in Figure 3a. Single-component substrates originating either from TOCNF or SDCtNF exhibited high transparency, whereas the TOCNF/SDCtNF composite substrate was a little bit translucent, possibly due to some aggregation. The surface wettability of cell culture scaffolds is a key issue to make an impact on cell attachment, and in general adequate hydrophobicity to promote the adsorption of adhesive proteins is required for practical cell culture [40]. The contact angles of a water droplet on each substrate, TOCNF alone, SDCtNF alone and TOCNF/SDCtNF composite, exhibited a hydrophilic surfaces, ranging from ca. 35° to ca. 50°, being regarded as a conventional bioinert surface, i.e., a non-cell-adhesive surface, as previously reported [41]. To determine the physical functionality of the substrates at a cell perception level, the surface roughness of the substrates was measured by AFM imaging. Figure 3b depicts the surface topography of each substrate, clearly indicating the accumulation of thin nanofibers to form dense network structures. The single-component TOCNF or SDCtNF substrates provided very flat surfaces with *R*a = 2.94 nm and 4.06 nm, respectively, while the TOCNF/SDCtNF composite showed relatively rough surface with *R*a = 53.4 nm, as shown in Table S1. This was attributed to the local aggregation induced by ionic crosslinking between negatively-charged carboxylates on the TOCNF and positively-charged amines on the SDCtNF [42,43], due to partial charge compensation presumed from the ζ-potential variation. Kunzler et al. reported that human gingival fibroblasts cultured on the same component substrate with different roughness at a micrometer level from 1 to 6 μm of *R*a exhibited different morphological behavior of cells during proliferation [44]. The TOCNF/SDCtNF composites having ca. 50 nm of *R*a were regarded as being very flat at the cell perception level. On the other hand, the nanometer-scale topography is considered to affect the intra-/intercellular sensing functions [38,39]. These polysaccharide NFs scaffolds possessed nanometer-scale roughness, and would possibly assume some influence on the cell behavior at the biointerfaces. Besides, the nanofibers-entangled structure was swollen but insoluble without adding any crosslinking agents in an aqueous medium during cell culture. NIH/3T3 fibroblast cells could proliferate on the surfaces of these nanofiber mats, which were much different from polymer films from a viewpoint of biointerface structures. However, the swollen structures of the nanofibers-entangled mats also may change the surface roughness in the culture medium, and therefore this concern will be investigated in our future work.

### 3.2. Proliferation Behavior of Mouse Fibroblasts on Polysaccharide NFs Substrates

**Figure 4:**
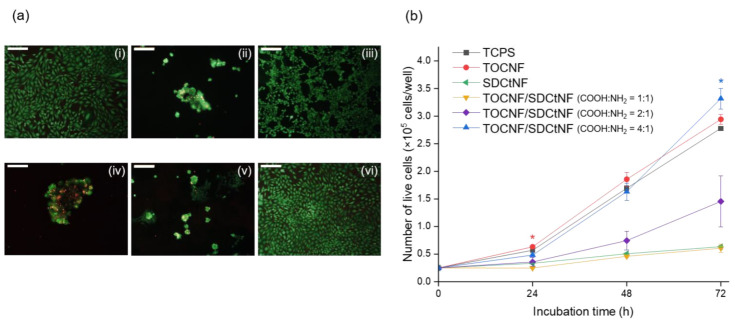
(**a**) Fluorescence images of NIH/3T3 cells cultured for 72 h of (**i**) TOCNF alone, (**ii**) SDCtNF alone, (**iii**) TCPS, (**iv**) TOCNF/SDCtNF (COOH:NH_2_ = 1:1), (**v**) TOCNF/SDCtNF (2:1) and (**vi**) TOCNF/SDCtNF (4:1). Live cells were stained with calcein AM (green) and dead cells with ethidium homodimer III (red). Scale bars: 200 μm. (**b**) Cell numbers on each substrate after 24, 48 and 72 h of culture. Mean ± SD, *n* = 3, * *p* < 0.05 vs. TCPS.

The effects of the combination of two polysaccharide NFs, TOCNF and SDCtNF, on cell viability and proliferation, were investigated using mouse fibroblast NIH/3T3 cells, which were subjected to cell culture either on single TOCNF, single SDCtNF or TOCNF/SDCtNF composite substrates. Figure 4a displays cell morphologies of NIH/3T3 cells with live/dead staining on each substrate after 72-h incubation. The NIH/3T3 cells adhered to and extended on the single TOCNF substrate as well as TCPS, reported in our previous study [29]; however, single SDCtNF substrate exhibited poor cell adhesion to form spheroid-like aggregates. The molar ratios of carboxylate and amine groups of the TOCNF/SDCtNF composites strongly influenced the cell growth behavior, as shown in Figure 4b and Figure S1. Increasing molar ratios of COOH:NH_2_ from 1:1 to 4:1 in the TOCNF/SDCtNF composite substrates markedly improved the cellular attachment, from spheroid formation to cell spreading after 72-h culture. The TOCNF/SDCtNF composite substrate with COOH:NH_2_ = 4:1 was superior to TCPS for cell proliferation, although polysaccharide NFs substrates exhibited hydrophilicity disadvantageous for conventional cell attachment. The surface charge modulates protein adsorption to direct integrin binding and specificity, thereby controlling cell adhesion. Thevenot et al. have reported that the incorporation of negative charges facilitated the adsorption of proteins which promoted cell adhesion and responses [45]. On the other hand, such strong interaction between material surfaces and cells made negative impacts on the cell growth rate due to strong cell adhesion. In this study, a single-component TOCNF substrate possesses a negative surface charge, ca. −46.8 mV of ζ-potential, possibly preferable for cell adhesion; however, it may interact strongly with the cultured cells. Thus, in the TOCNF/SDCtNF composite substrates, mixing TOCNF and SDCtNF having opposite charges, as shown in Table S1, was presumably allowed to tune the surface characteristics for cell adhesion and subsequent proliferation.

The amine content of SDCtNF used here was 1.71 mmol g^–1^, which was the maximum value as reported [19]; however, further *N*-deacetylation inside of CtNF was possible to form highly-deacetylated CsNF. Thus, another combination of TOCNF and commercial CsNF (NH_2_: 4.35 mmol g^–1^) was investigated as comparison. TEM image of CsNF exhibited thicker fibrous morphology than SDCtNF, and thin TOCNFs were entangled around the thick CsNF in the composite (Figure S2a). CsNF showed a typical XRD pattern of Cs; but the crystallinity was relatively low (Figure S2b). The surface roughness (*R*a) of the CsNF substrate was much greater than SDCtNF and TOCNF (Figure S3). Therefore, the uniform substrates composed of TOCNF and CsNF could not be obtained by simple mixing. Nevertheless, molar ratios of carboxylate and amine of TOCNF/CsNF composites strongly affected the proliferation rate of NIH/3T3 cells (Figure S4). In this case, increasing molar ratios of COOH:NH_2_ tended to decrease cell proliferation efficiency, where the COOH:NH_2_ = 1:1 was the most preferable for cell growth (Figure S5). Direct comparison was a little difficult because the amine content of CsNF was 2.5 times greater than that of SDCtNF. The negatively-charged TOCNF possibly interacted with the outer surface of positively-charged NFs. The weight ratio of TOCNF and SDCtNF at COOH:NH_2_ = 4:1 was almost the same as that of TOCNF and CsNF at COOH:NH_2_ = 1:0.63, possibly assuming the similar situation for TOCNF-accessible amines exposed on the surfaces of SDCtNF and CsNF.

An important thing here was the fact that the simple molecular blend of CMC and Cs made no contribution to such unique cell proliferation behavior even at the molar ratios of COOH:NH_2_ = 1:1 and 4:1, as shown in Figure S6. Although CMC and Cs had carboxylate and amine groups, respectively, similar as TOCNF and SDCtNF, the combination of CMC and Cs was not effective at all for cell culture, strongly indicating the significance of nanofiber forms in order to enhance the cell proliferation behavior. In vivo, the ECM biocomponents in general possess the nanofibrous forms, not molecular ones; therefore, a lot of research on cell culture substrates has paid attention to various synthetic and natural nanofibers [46,47]. The nanofibers-entangled structure was swollen but insoluble without any crosslinkers in an aqueous medium. NIH/3T3 fibroblast cells could proliferate on the surfaces of these nanofiber mats. It is not the purpose of this study to achieve complete imitation of natural ECM structures by using TOCNF and SDCtNF. Our polysaccharide NFs possessed solid-state interfaces, possibly interacting with the adhered cells at the nanometer level via stimulating intra-/intercellular sensing functions [38,39]. In this study, we have proposed to use TOCNF and SDCtNF, which both possess very fine, stable, natural-origin and xeno-free nanofiber architecture, and the combination of negatively-charged TOCNF and positively-charged SDCtNF is expected to tune the surface physicochemistry of the scaffold, directly affecting cell culture behavior.

### 3.3. Cell Migration Behavior for Wound Healing

**Figure 5:**
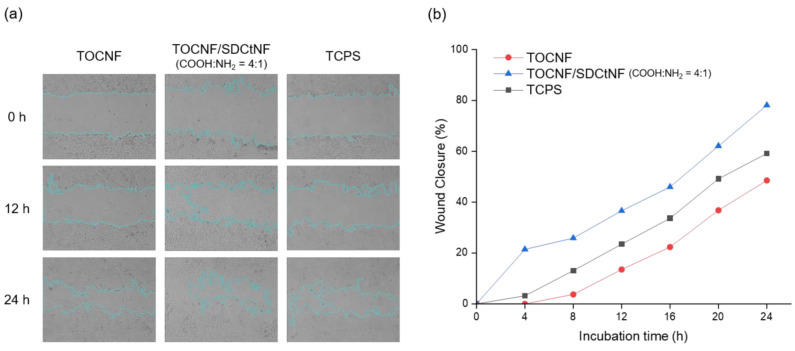
(**a**) Representative snapshot images of wound closure behavior of NIH/3T3 cells on different substrates. Cyan lines indicate the edges of the moving cells during the migration. (**b**) Wound closure rate of NIH/3T3 cells cultured on different substrates.

Cell migration is an essential process for multicellular organisms, and indispensable for tissue developments, repair and regeneration [48]. Directional migration is in general triggered in response to extracellular stimuli such as chemokines and ECM components [49,50]. As found out above, the combination of TOCNF and SDCtNF could control the cell adhesion and proliferation behavior; therefore it was expected to manipulate cell migration. Thus, wound healing assay was investigated using mouse NIH/3T3 cells on our substrates. Figure 5a shows snapshot images of cell migration during wound closure of cell sheets with a gap of 500 µm fabricated on each substrate, and wound closure rates are shown in Figure 5b. Video S1 visualizes wound repair behavior for 48 h with animation of the obtained snapshots. TOCNF/SDCtNF substrate remarkably accelerated the wound closure process in the NIH/3T3 cell culture. Cell growth on the single-component SDCtNF substrate could not reach to be confluent, resulting in much difficulty in cell migration test (data not shown). The wound closure percentage on single TOCNF substrate varied from 13.5% for 12 h to 48.6% for 24 h, as compared to an initial area at t = 0, whereas control TCPS substrate exhibited the slow closure varying from 23.4% for 12 h to 60.6% for 24 h. Thus, it was presumably indicated that the NIH/3T3 cells strongly adhered to the surface of TOCNF rather than TCPS, resulting in less migration. On the other hand, the combination of TOCNF and SDCtNF with a molar ratio of COOH:NH_2_ = 4:1 demonstrated rapid wound closure varying from 36.6% for 12 h to 78.1% for 24 h, presumably indicating the promotion of cell migration. Wound closure rate was faster for the combination of TOCNF and SDCtNF substrate than TOCNF alone and TCPS as control. Although single-component TOCNF substrate was effective for promoting the strong adhesion of NIH/3T3 fibroblasts to the scaffold surface, such interaction was not effective for cell migration, resulting in slow wound repair. Water-soluble Cs molecules have been investigated for fibroblast activation, cytokine production and stimulation of type IV collagen synthesis [51]; however poor cell adhesion causes problems for in vitro cell culture. Our strategy for the combination of natural polysaccharide nanofibers, TOCNF and SDCtNF, allowed to promote cell attachment, subsequent cell proliferation and smooth cell migration, which is expected to expand the possibility to tune the physicochemical and biological properties of xeno-free cell culture scaffolds.

## 4. Conclusions

In conclusion, the combination of two types of polysaccharide nanofibers, TOCNF and SDCtNF, was effective for modulating cell attachment, subsequent proliferation and rapid cell migration due to the soft attachment of mouse fibroblast NIH/3T3 cells to the substrates. Fibroblast proliferation strongly depended on the molar ratios of functional groups, carboxylates and amines, respectively, present on TOCNF and SDCtNF surfaces. Molecular blend of water-soluble polysaccharides, analogous to TOCNF and SDCtNF, exhibited no positive effect on such unique cellular behavior. Thus, rigid nanofiber forms like ECM biocomponents found in vivo must be a key issue to manipulate the cellular response. Unique combination of structural natural polysaccharide nanofibers would be a promising strategy to design bioadaptive nanomaterials for tissue engineering.

## Data Availability

Data presented in this study are available in this article.

## Supplementary Materials

The following supporting information can be downloaded at: https://www.mdpi.com/article/10.3390/nano12030402/s1, Table S1: Characterization of polysaccharide NFs and substrates used in this study; Figure S1: Optical and fluorescence images of NIH/3T3 cells cultured on each substrate for 24, 48 and 72 h; Figure S2: TEM images of CsNF and TOCNF/CsNF (COOH:NH_2_ = 1:1); XRD pattern of CsNF; Figure S3: Characterization of polysaccharide NFs-coated substrates; Optical and AFM images of CsNF alone and TOCNF/CsNF composite (COOH:NH_2_ = 1:1); Contact angles of a water droplet on each substrate after sterilization; Figure S4: Optical and fluorescence images of NIH/3T3 cells cultured on TOCNF/CsNF substrate for 24, 48 and 72 h; Figure S5: Cell proliferation behavior on TOCNF/CsNF substrates; Time-course profiles of cell growth on TOCNF alone, CsNF alone and TOCNF/CsNF composite (COOH:NH_2_ = 1:1); Effect of molar ratios of COOH and NH_2_ in the TOCNF/CsNF composite substrates on cell proliferation at 72 h; Figure S6: Optical and fluorescence images of NIH/3T3 cells cultured on CMC/Cs substrates for 24, 48 and 72 h; Video S1: Wound closure behavior of NIH/3T3 cells on each substrate; TOCNF alone, TOCNF/SDCtNF composite (COOH:NH_2_ = 4:1) and TCPS.
