# Supplementary material for: Combination of Polysaccharide Nanofibers Derived from Cellulose and Chitin Promotes the Adhesion, Migration and Proliferation of Mouse Fibroblast Cells
Source: Nanomaterials (Basel). 2022 Jan 26;12(3):402. doi: 10.3390/nano12030402 (PMC8840717; doi:10.3390/nano12030402)
Supplement: Supplementary file 1 [file nanomaterials-12-00402-s001.zip › nanomaterials-1551543-supplementary.pdf]

nanomaterials

Supporting Information

Combination of Polysaccharide Nanofibers Derived from Cellulose and Chitin Promotes the Adhesion, Migration and Proliferation of Mouse Fibroblast Cells

Tomoka Noda, Mayumi Hatakeyama and Takuya Kitaoka *

Department of Agro-Environmental Sciences, Graduate School of Bioresource and Bioenvironmental Sciences, Kyushu University, Fukuoka 819-0395, Japan; t.noda@agr.kyushu-u.ac.jp (T.N.); m_hatakeyama@agr.kyushu-u.ac.jp (M.H.)

* Correspondence: tkitaoka@agr.kyushu-u.ac.jp; Tel.: +81-92-802-4665

Table of Contents

1. Characterization of polysaccharide nanofibers and substrates
2. Optical and fluorescence images of NIH/3T3 cells cultured on TOCNF, SDCtNF and TOCNF/SDCtNF substrates
3. Characterization of CsNF and CsNF substrates
4. Cell culture using TOCNF/CsNF composite substrates
5. Cell culture using CMC/Cs molecular blend substrates
6. Reference

Citation: Noda, T.; Hatakeyama, M.; Kitaoka, T. Combination of Polysaccharide Nanofibers Derived from Cellulose and Chitin Promotes the Adhesion, Migration and Proliferation of Mouse Fibroblast Cells. *Nanomaterials* **2022**, *12*, 402. https://doi.org/10.3390/nano12030402

Academic Editor: Wei Zhang

Received: 27 December 2021
Accepted: 21 January 2022
Published: 26 January 2022

Publisher's Note: MDPI stays neutral with regard to jurisdictional claims in published maps and institutional affiliations.

Copyright: © 2022 by the authors. Licensee MDPI, Basel, Switzerland. This article is an open access article distributed under the terms and conditions of the Creative Commons Attribution (CC BY) license (https://creativecommons.org/licenses/by/4.0/).

1. Characterization of polysaccharide nanofibers and substrates

Atomic force microscopy (AFM, Dimension Icon, Bruker AXS GmbH, Germany) was carried out to determine the width of TOCNF and SDCtNF from the height profiles of the corresponding 50 single nanofibers observed in the AFM images. The ζ-potential values of TOCNF, SDCtNF and TOCNF/SDCtNF mixtures in water were measured at pH 7.0 using a Zetasizer Nano ZS instrument equipped with a 633-nm HeNe laser operating at a 173° detector angle (Malvern Panalytical Ltd., Tokyo, Japan). Aqueous dispersions of TOCNF and SDCtNF for the ζ-potential measurement were prepared at 0.03 wt% and 0.02 wt%, respectively. TOCNF/SDCtNF mixtures were prepared by blending each aqueous dispersion according to the molar ratios of COOH:NH_2.

Table S1. Characteristic data of the nanofibers and substrates used in this study.

Sample	Nanofiber		Substrate		
	**Fiber width (nm)**	**ζ-Potential [1] (mV)**	**Fiber content (mg/well)**		***R*a (nm) [2]**
			**TOCNF**	**SDCtNF**	
TOCNF	2.33 ± 0.91	–46.82 ± 4.17	0.80	-	2.94
SDCtNF	4.89 ± 2.12	17.52 ± 0.76	-	0.80	4.06
TOCNF/SDCtNF (COOH:NH_2 = 1:1)	-	–16.14 ± 3.26	0.41	0.39	38.9
TOCNF/SDCtNF (COOH:NH_2 = 2:1)	-	–36.43 ± 3.41	0.55	0.25	57.1
TOCNF/SDCtNF (COOH:NH_2 = 4:1)	-	–39.95 ± 0.68	0.65	0.15	53.4

[1] Measured at n = 3 for each sample. [2] Root mean square roughness (*R*a) was calculated from the AFM images of each substrate's surface (scanning range: 20 × 20 μm^2, see Section 2.3 in the main text).

2. Optical and fluorescence images of NIH/3T3 cells cultured on TOCNF, SDCtNF and TOCNF/SDCtNF substrates

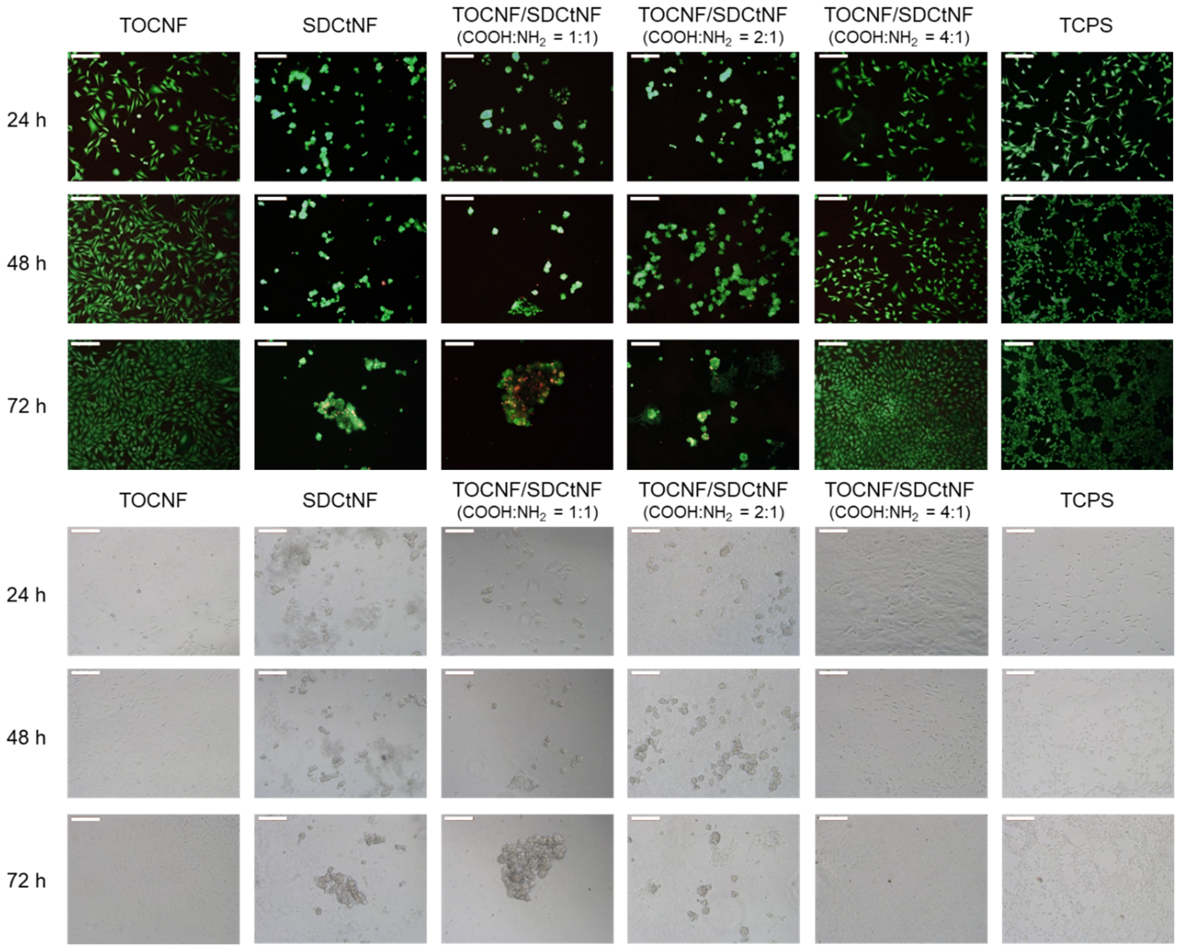

Figure S1. Optical (upper) and fluorescence (lower) images of NIH/3T3 cells cultured on each substrate for 24, 48 and 72 h. Scale bars = 200 μm. The fluorescence images at 72 h are the same as in Figure 4 in the main text.

3. Characterization of CsNF and CsNF substrates.

Chitosan nanofiber (CsNF) was purchased from Sugino Machine Limited, Uozu, Japan (BiNFi-s, EFo-08002, 2.0 wt%, NH_2: 4.35 mmol g^{-1}). The nanomorphologies of CsNF and TOCNF/CsNF mixture (COOH:NH_2 = 1:1) were observed by TEM (Figure S2a). The crystalline structure of CsNF was characterized by XRD (Figure S2b). The width of CsNF was *ca.* 26.12 nm from the TEM images (n = 50). TEM imaging shows that CsNF exhibited a nanofiber shape; however, its fiber width was much larger than those of TOCNF and SDCtNF, as shown in Figure 2 and Table S1. Thus, the mixture of TOCNF and CsNF depicts thin TOCNFs entangled around the thick CsNF (Figure S2a (ii)). The XRD pattern shows the typical crystalline structure of chitosan [1], and the crystallinity index was *ca.* 67.8%. Polysaccharide NFs-casted substrates were prepared using aqueous dispersions of 0.4 wt% TOCNF and 0.3 wt% CsNF, in the same manner as for TOCNF/SDCtNF substrates (see Section 2.2 in the main text). The appearance of single-component CsNF and TOCNF/CsNF composite substrates was slightly cloudy (Figure S3a). The surface morphology was evaluated with AFM analysis (Figure S3b). The surface roughness (*R*a) was 77.2 nm for CsNF alone and 73.0 nm for TOCNF/CsNF composite (COOH:NH_2 = 1:1).

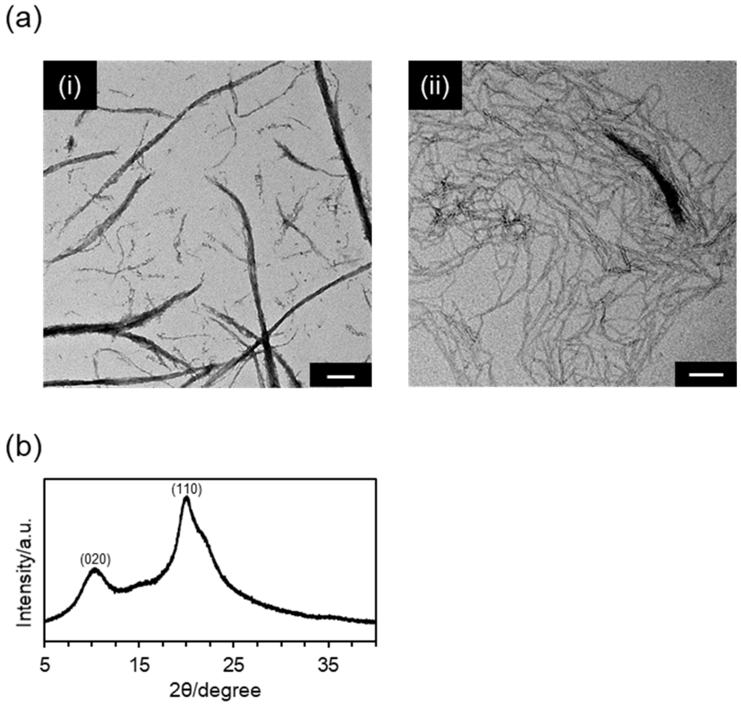

Figure S2. TEM images (a) of (i) CsNF and (ii) TOCNF/CsNF (COOH:NH_2 = 1:1). Scale bars in (a) = 200 nm. XRD pattern (b) of CsNF.

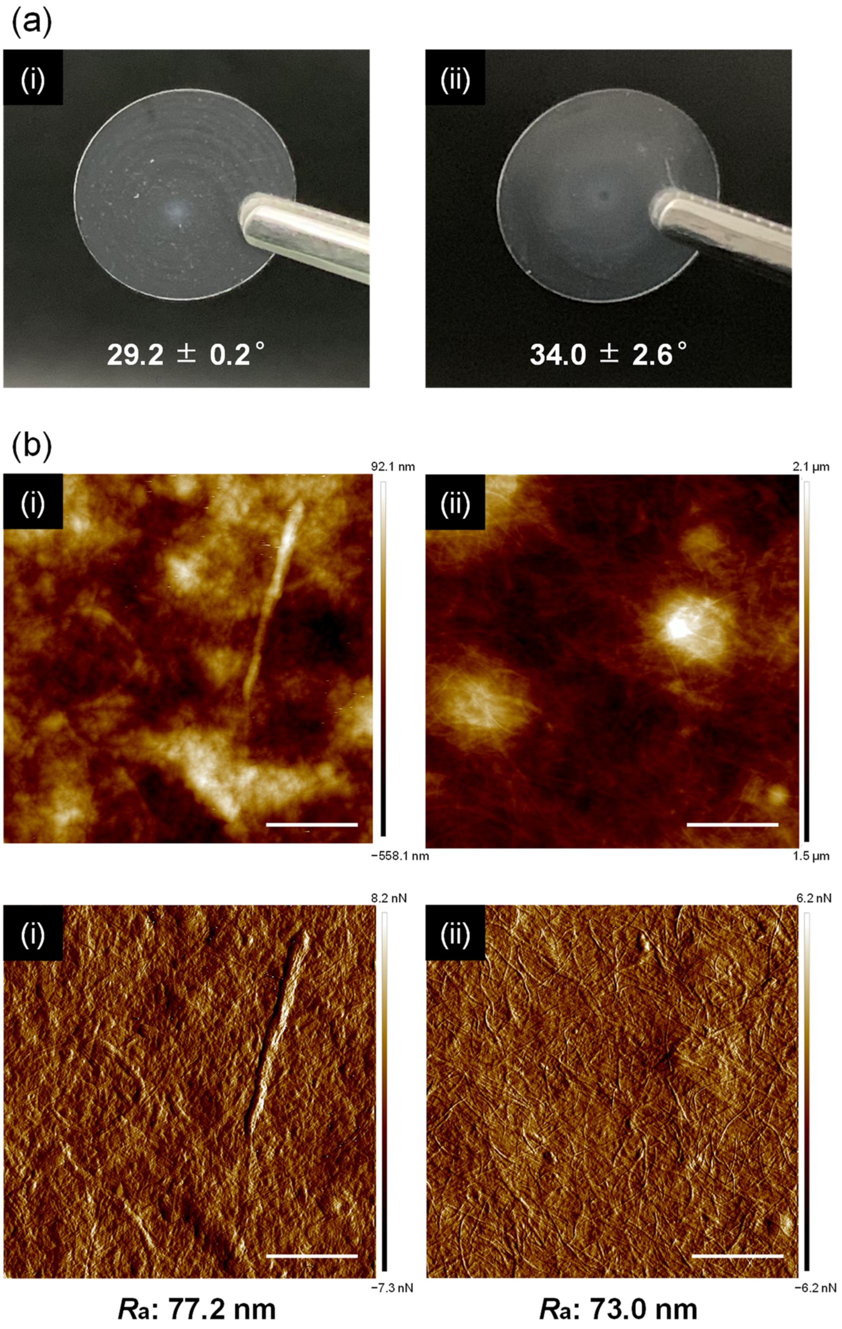

Figure S3. Characterization of polysaccharide NFs-coated substrates. Optical (a) and AFM (b) images of (i) CsNF alone and (ii) TOCNF/CsNF composite (COOH:NH_2 = 1:1). Contact angles of a water droplet on each substrate after sterilization are shown in (a). Upper and lower images of (b) correspond to the topological and peak force error images, respectively. Scale bars in (b) = 5 μm.

4. Cell culture using TOCNF/CsNF composite substrates

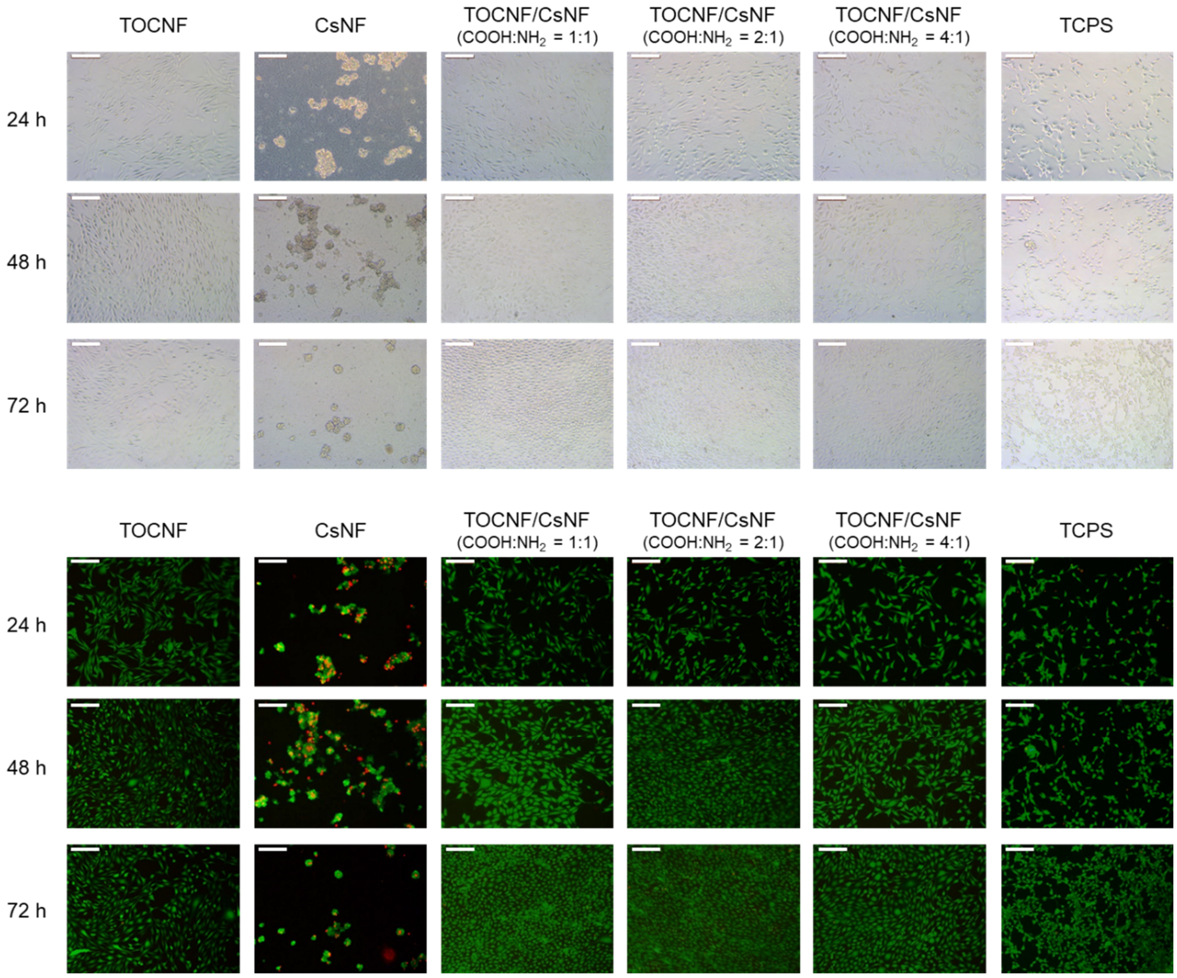

Figure S4. Optical (upper) and fluorescence (lower) images of NIH/3T3 cells cultured on TOCNF/CsNF substrate for 24, 48 and 72 h. Scale bars = 200 μm.

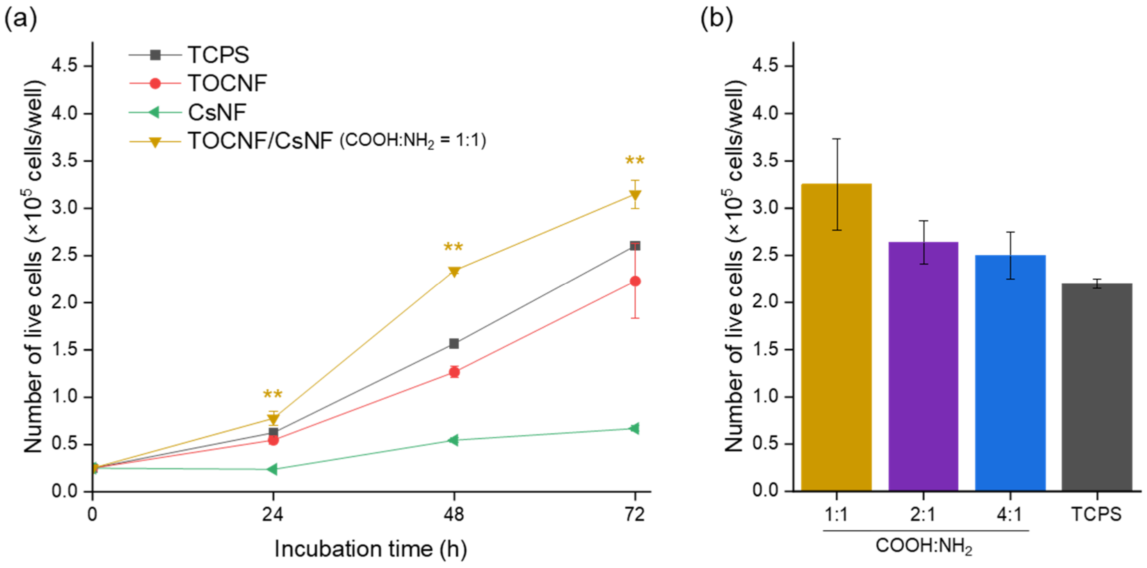

Figure S5. Cell proliferation behavior on TOCNF/CsNF substrates. (a) Time-course profiles of cell growth on TOCNF alone, CsNF alone and TOCNF/CsNF composite (COOH:NH_2 = 1:1). (b) Effect of molar ratios of COOH and NH_2 in the TOCNF/CsNF composite substrates on cell proliferation at 72 h. Mean ± SD, $n = 3$, $^{**}p < 0.01$ *vs* TCPS.

5. Cell culture using CMC/Cs molecular blend substrates.

Carboxymethylcellulose (CMC, COOH: 2.24 mmol g^{-1}) and chitosan powder (Cs, fully *N*-deacetylated, NH_2: 6.82 mmol g^{-1}) were purchased from FUJIFILM Wako Pure Chemical Corp., Osaka, Japan. CMC was dissolved in ultrapure water to make 0.4 wt% solution. Cs powder was dissolved in 0.1 mol L^{-1} aqueous acetic acid solution to a concentration of 0.4 wt%. Molecular polysaccharides-casted substrates were prepared in the same manner as for TOCNF/SDCtNF substrates (see Section 2.2 in the main text). As-prepared substrates were thoroughly washed three times with phosphate buffered saline without divalent ions, PBS. The dried substrates were sterilized by immersion in ethanol with UV light for 20 min.

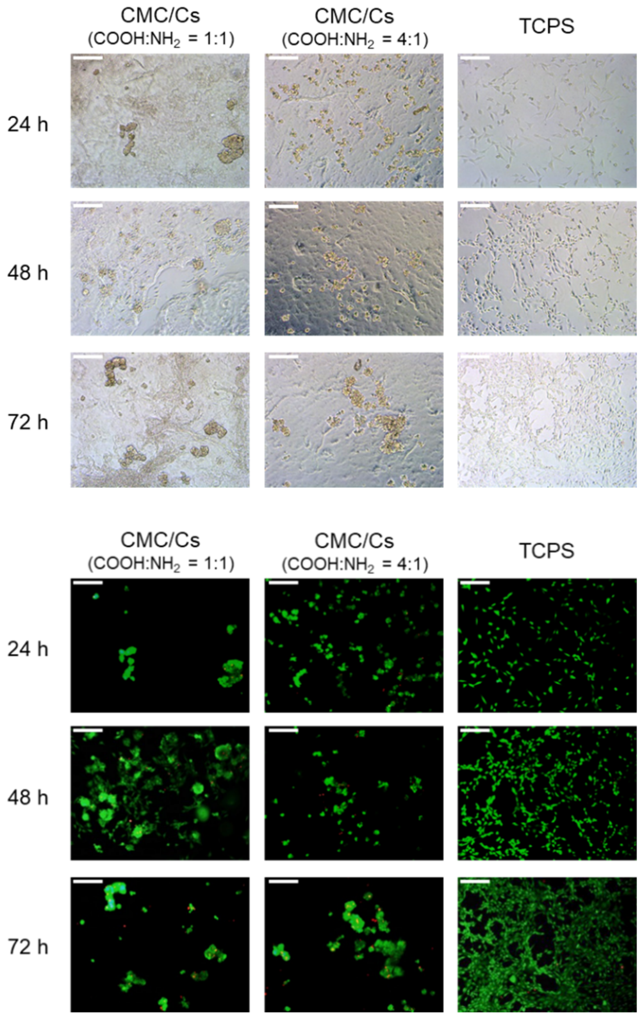

Figure S6. Optical (upper) and fluorescence (lower) images of NIH/3T3 cells cultured on CMC/Cs substrates for 24, 48 and 72 h. Scale bars = 200 μm.

Reference

1. Zhang, Y.; Xue, C.; Xue, Y.; Gao, R.; Zhang, X. Determination of the degree of deacetylation of chitin and chitosan by X-ray powder diffraction. *Carbohydr. Res.* **2005**, *340*, 1914–1917, doi:10.1016/J.CARRES.2005.05.005.
